# Risk model and factors for prediction of response to neoadjuvant chemotherapy in patients with advanced gastric cancer-a two-center cohort study

**DOI:** 10.1186/s12885-023-10513-1

**Published:** 2023-01-11

**Authors:** Xian-Wen Liang, Wei-Sheng Xiao, Hao Lei, Qian-Cheng Huag, Yu-Lan Dong, Fang Wang, Wei-Peng Qing

**Affiliations:** 1 Present Address: Department of Hepatobiliary Surgery, Hainan General Hospital, Haikou, China; 2 Department of Gastrointestinal Surgery, Central South University Xiangya School of Medicine Affiliated Haikou Hospital, Haikou, China; 3 Gastroenterology, The First Affiliated Hospital of Hengyang Medical College, University of South China, Hengyang, China; 4 Radiology Department, The First Affiliated Hospital of Hengyang Medical College, University of South China, Hengyang, China

**Keywords:** Advanced gastric cancer, Predictor, Neoadjuvant chemotherapy, Nomogram, RECIST

## Abstract

**Objective:**

Due to inconsistency in neoadjuvant chemotherapy (NACT) response in advanced gastric cancer (GC), the indications remain the source of controversy. This study focused on identifying factors related to NACT chemosensitivity and providing the best treatment for GC cases.

**Methods:**

Clinical data in 867 GC cases treated with neoadjuvant chemotherapy were downloaded from two medical centers between January 2014 and December 2020, and analyzed by logistic regression and the least absolute shrinkage and selection operator (LASSO) for identifying potential factors that predicted NACT response and might be incorporated in constructing the prediction nomogram.

**Results:**

After the inclusion and exclusion criteria were applied, totally 460 cases were enrolled, among which, 307 were males (66.74%) whereas 153 were females (33.26%), with the age of 24–77 (average, 59.37 ± 10.60) years. Consistent with RECIST standard, 242 patients were classified into effective group (PR or CR) while 218 were into ineffective group (PD or SD), with the effective rate of 52.61%. In training set, LASSO and logistic regression analysis showed that five risk factors were significantly associated with NACT effectiveness, including tumor location, Smoking history, T and N stages, and differentiation. In terms of our prediction model, its C-index was 0.842. Moreover, calibration curve showed that the model-predicted results were in good consistence with actual results. Validation based on internal and external validation sets exhibited consistency between training set results and ours.

**Conclusions:**

This study identified five risk factors which were significantly associated with NACT response, including smoking history, clinical T stage, clinical N stage, tumor location and differentiation. The prediction model that exhibited satisfying ability to predict NACT effectiveness was constructed, which may be adopted for identifying the best therapeutic strategy for advanced GC by gastrointestinal surgeons.

## Introduction

Gastric cancer (GC) ranks the fourth place among cancers with regard to the mortality [1], which causes approximately 770,000 deaths annually, making it the fifth cause leading to cancer-associated mortality globally [2]. However, GC lacks early symptoms, which also adds to the difficulty in early diagnosis. The 5-year survival for advanced GC patients is just 25–31% [3–6]. Although gastrectomy combined with D2 lymph node dissection (LND) and postoperative chemotherapy can increase advanced GC patient survival, their overall survival (OS) remains poor. Recently, in order to improve its efficacy in advanced GC patients, neoadjuvant chemotherapy (NACT) has been proposed to be the important treatment by some national and international guidelines [7]. NACT mainly aims to achieve tumor downstaging and provide the possibility of R0 resection for advanced GC cases [8]. The 2019 National Comprehensive Cancer Network (NCCN) guidelines recommended that NACT should be considered in cases whose clinical TNM stage is ≥ T2N [9]. It is recommended by Japanese treatment guidelines (5th edition) that, NACT should be performed in cases of T2-T4 stage and lymph node enlargement [10].

NACT can decrease the tumor loading, down-stage tumor, and increase radical resection ratio while enhancing patient life quality; nonetheless, some controversial points still exist, including indications, frequency and scheme for chemotherapy [11]. As reported in some studies, survival benefits of NACT are dependent on chemotherapeutic response of tumor, indicating that patients who achieve complete pathological response to NACT may obtain long OS and disease-free survival (DFS) [12–14], whereas patients who achieve poor chemotherapeutic response and no significantly reduced tumor following NACT may have dismal survival. In cases who achieve the poor objective response to NACT, NACT will delay the operation date and induce severe toxic adverse reactions in patients. Therefore, it is of great significance to estimate GC response to NACT and evaluate the suitability of GC patients for NACT. Comprehensive treatment or surgery must be performed early for insensitive patients. In recent studies, greater efforts have been made to discover the predicting factors for NACT response, and nomogram models are constructed to predict the prognosis of advanced GC following NAC [15–19]. Compared with conventional segmented models, the NACT nomograms are superior.

Although many studies discuss patient prognosis and NACT-related postoperative complications, only a small proportion of them have identified predicting factors for NACT efficacy prior to chemotherapy.

Therefore, this retrospective study focused on examining clinical parameters and tumor biological characteristics affecting the NACT efficacy among advanced GC cases, and constructing the prediction nomogram for predicting NACT effectiveness, aiming to offer personalized treatments and provide the most benefits for advanced GC patients.

## Materials and methods

### Patients and data extraction

**Fig. 1:**
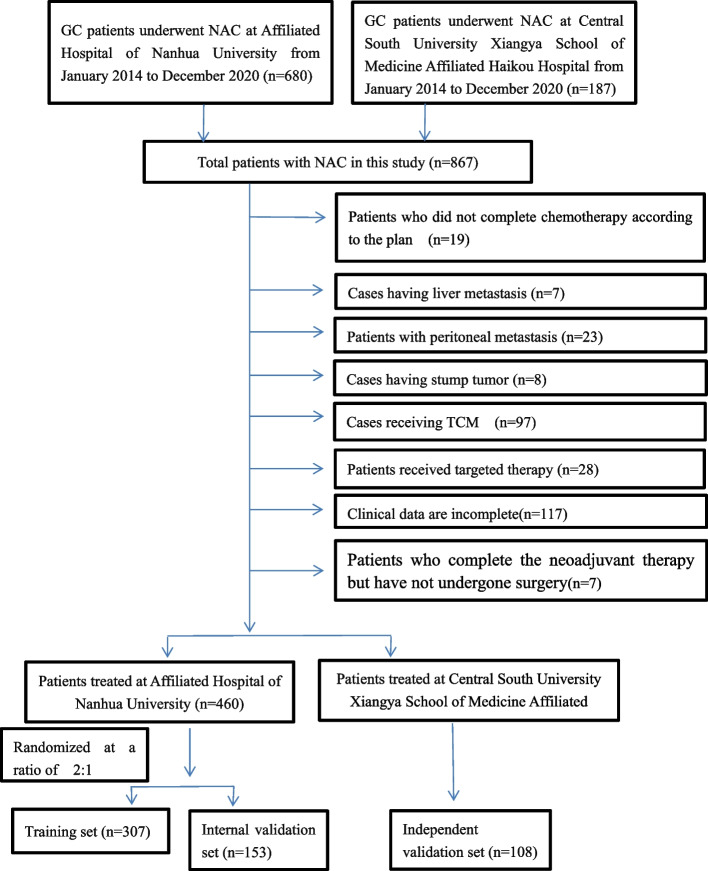
Flow diagram showing GC case selection process

Patient records were anonymized and de-identified before the analyses. The present retrospective study was approved by Research Ethics Committee of Affiliated Hospital of University of South China and The Central South University Xiangya School of Medicine Affiliated Haikou Hospital. Clinical information was extracted in medical records of 867 advanced GC cases receiving NACT at The First Affiliated Hospital of University of South China and The Central South University Xiangya School of Medicine Affiliated Haikou Hospital from January 2014 to December 2020. Then, the extracted information was subject to retrospective analysis. Patients conforming to the following criteria were enrolled: ① cases with the diagnosis of GC via biopsy or gastroscopy; ② clinical stage of GC cases was T2N + M0 or T3-4N0 / + M0; ③ cases completing NAC; ④ GC cases underwent radical gastrectomy following NACT; ⑤ cases receiving Capecitabine plus oxaliplatin (XELOX) as the chemotherapy regimen; and ⑥ cases aged 18–80 years. Patients conforming to the criteria below were excluded: ① patients who did not complete chemotherapy according to the plan (18 people stopped chemotherapy early because of severe adverse reactions, and one patient discontinued treatment because he did not agree to continue chemotherapy); ② cases who had additional cancers; ③ cases who had gastric stump tumor; ④ cases receiving additional anticancer therapies including radiotherapy or traditional Chinese medicine (TCM), and ⑤ cases with insufficient clinical information. Finally, totally 568 patients were enrolled into the present study. To be specific, 460 of these cases treated in The First Affiliated Hospital, University of South China were randomized into training and internal validation sets (2:1). Meanwhile, 108 patients from The Central South University Xiangya School of Medicine Affiliated Haikou Hospital were classified into the external validation set. Figure 1 shows the process of patient inclusion.

### Treatment

Laparoscopic exploration was performed among cases at T2N + M0 or T3-4N0 / +M0 stage. Tumor was removed when no additional distant metastasis was discovered, like intraperitoneal space metastasis, followed by 3 cycles of chemotherapy on day 1 following laparoscopic exploration. The chemotherapy regimen was Capecitabine plus oxaliplatin (XELOX). Dosage was adjusted according to patient tolerance and effectiveness. At 2 weeks post-NACT, enhanced CT or endoscopy was conducted to confirm whether the primary tumor was resectable, followed by implementation of surgery. Each of our included cases underwent radical surgical resection (open/laparoscopic surgery, total/subtotal gastrectomy) in combination with D2 lymphadenectomy.

Toxicity was evaluated according to the National Cancer Institute’s Common Terminology Criteria for Adverse Events, version 4.0. In this study, 18 people stopped chemotherapy early because of severe adverse reactions, and the overall incidence of NAC adverse events was 82.34%. The rate of grade 3/4 toxicity was 32.78%. The main side effects were hematological toxicity and gastrointestinal reaction. Anemia was the most common grade 3/4 adverse event.

### Data extraction

Baseline clinical information was collected prior to NACT, including sex, age, and smoking history (In line with the WHO guidelines, smoking history was deemed as patients with continuous or cumulative smoking for at least 6 months; otherwise, the patient did not have a smoking history), blood group, BMI, tumor size, tumor location, tumor markers (CA125, CA199,CA742, CEA), infiltration depth, lymph node metastasis (LNM), Borrmann classification, pathological classification, microsatellite instability (MSI), albumin, lymphocytes, platelet count, monocytes, and neutrophils. Meanwhile, tumor size, infiltration depth as well as LNM was evaluated by laparoscopic exploration combined with enhanced CT prior to NACT. The efficacy assessment standard for neoadjuvant chemotherapy was evaluated according to the solid tumor response evaluation (RECIST) standard proposed by Therasse et al. in 2000 [20]. Taking into account the measurement of the maximal diameter only for all target lesions: complete response (CR) was deemed as no visible tumors in each target lesion; partial response (PR) was deemed as that compared with the baseline, markedly decreased total longest diameter for all tumors (≥30%), progressive disease (PD) was interpreted as at least one novel lesion or over 20% increase in target lesions maximal diameter [20], with the lowest total longest diameter measured during the treatment course being control; stable disease (SD) was intermediate between those of partial response and progressive. CR or PR was classified as the effective group, SD or PD as the ineffective group.

### Statistical analysis

R version 4.0.3 software (The R Foundation for Statistical Computing, Vienna, Austria. www. r-project. org) with the Hmisc, lattice, survival, Formula, ggplot2, SparseM, Matrix, rms, rmda packages and SPSS22.0 (IBM, Armonk, NY, United States) were employed for statistical analyses.

#### Univariate analysis

**T**he Kolmogorov-Smirnov test (K-S test) was conducted to verify the normality assumption, and factors with non-normal distribution were presented as median (25% IQR-75% IQR) and examined through Mann-Whitney test. Moreover, factors with normal distribution were displayed as mean ± SD and compared through Student’s T-test. Additionally, chi-square test was applied in analyzing categorical variables. α = 0.05 was the significance level.

#### Multivariable analysis

Statistically significant variables for outcomes of neoadjuvant chemotherapeutic response was analyzed by logistic regression (*P* < 0.05), while the least absolute shrinkage and selection operator (LASSO) regression was conducted for selecting the most useful predictive factors. We predicted odds ratio (OR), 95% confidence intervals (CIs) and regression coefficients.

#### Nomogram establishment

For predicting NACT response, the glm R package (version 4.0.3) was used to establish the nomogram that incorporated factors with prognostic significance upon logistic regression. After calculating the consistency index (CI), the correction curves were plotted and decision curve analysis (DCA) was conducted for evaluating nomogram’s prediction performance.

## Results

Table 1 displays baseline patient features. All cases were randomized into training (*n* = 307) or internal validation (*n* = 153) set by R package “set, seed” function, as shown in Table 1. Using the inclusion and exclusion criteria, totally 460 cases were enrolled into this study. Among them, 307 were males (66.74%) and 153 were females (33.26%), with the age of 24–77 (average, 59.21 ± 10.16) years. In training set, based on the RECIST standard, 162 patients were in effective group (PR 38 and CR 124) whereas 145 were in ineffective group (PD 104 and SD 41). The effective rate was 52.77%. There were 95 patients of T2 or T3 infiltration depth, while 212 of T4 stage. Moreover, 111 cases (36.16%) had lesions at the esophagogastric junction. In addition, 240 cases had positive LNM, occupying 78.18%. After completing NACT, T staging decreased in 141 (45.93%) and increased in 62 (20.20%), while N stage decreased in 120 patients (39.08%) and increased in 22 patients (7.17%).

**Table 1:** Baseline characteristics of included gastric cancer patients

Variable		Training set (*n* = 307)	Validation set (*n* = 153)	P
Age		59.18 ± 10.12	59.22 ± 10.46	0.802
Sex	Male	207 (67.43)	100 (65.36)	0.657
	Female	100 (32.57)	53 (34.64)	
BMI		22.38 (20.51–24.91)	22.47 (20.18–24.54)	0.712
Location	Esophagogastric junction	108 (35.18)	58 (37.91)	0.566
	Non-Esophagogastric junction	199 (64.82)	95 (62.09)	
Tumor size, cm		5.38 ± 2.07	5.71 ± 3.02	0.478
Tumor differentiation	Well+Moderately differentiated	111 (36.16)	55 (35.95)	0.965
	Poorly differentiated+ Signet ring cell	196 (63.84)	98 (64.05)	
cT stage	T2 + T3	97 (31.60)	45 (29.41)	0.633
	T4	210 (68.40)	108 (70.59)	
cN stage	N0	60 (19.54)	40 (26.14)	0.106
	N+	247 (80.46)	113 (73.86)	
Borrmann classification	I + II	88 (28.66)	44 (28.76)	0.983
	III + IV	219 (71.33)	109 (71.24)	
Blood type	Type A	88 (28.66)	46 (30.07)	0.883
	Type B	90 (29.32)	42 (27.45)	
	Type AB	30 (9.77)	18 (11.76)	
	Type O	99 (32.25)	47 (30.72)	
CEA, ng/mL		9.14 ± 10.12	10.21 ± 11.37	0.375
CA724, U/mL		3.61 (1.57–10.52)	3.55 (1.50–10.33)	0.446
CA125, U/mL		13.96 ± 7.03	13.94 ± 7.22	0.743
CA199, U/mL		37.64 ± 42.18	38.05 ± 57.32	0.556
Albumin, g/L		42.13 ± 3.08	41.97 ± 3.77	0.289
PLT, 10^9^/L		218.76 ± 28.40	214.49 ± 52.78	0.472
Lymphocyte, 10^9^/L		1.58 ± 0.42	1.58 ± 0.46	0.943
PLR		135.76 (94.88–182.89)	136.27 (104.42–182.73)	0.575
Neutrophil cell, 10^9^/L		3.60 ± 1.31	3.61 ± 1.44	0.667
Monocyte, 10^9^/L		0.42 ± 0.15	0.41 ± 0.17	0.782
NMR		8.62 (6.62–10.64)	8.58 (6.50–10.31)	0.455
NLR		2.24 (1.58–2.94)	2.24 (1.60–2.89)	0.889
MSI	H	21 (6.84)	12 (7.84)	0.695
	S/L	286 (93.16)	141 (92.16)	
Smoking history	yes	110 (35.83)	60 (39.22)	0.479
	no	197 (64.17)	93 (60.78)	

Table 2 displays univariable relations of clinical factors with NACT effectiveness. Factors with statistical significance (*P* < 0.05) included tumor location, size, Clinical T stage, Clinical N stage, differentiation, and Smoking history. Thus, a higher response was observed in tumor located at the esophagogastric junction compared with that at the non-esophagogastric junction. In addition, the greater NACT response was observed in patients with lower tumor volume, higher differentiation degree (well/moderate vs. low differentiation), no lymph node metastasis, lower T stage (T2/T3 vs. T4 stage), and no smoking history.

**Table 2:** Characteristics of Patients in the training set and *P* value of univariate analysis. Factors with statistical significance include tumor location, size, Clinical T stage, Clinical N stage, differentiation, Smoking history

Characteristics		effective group (CR/PR)*n* = 162(%)	ineffective group (PD/SD)*n* = 145(%)	t/χ2	P
Age		59.64 ± 10.02	59.06 ± 11.25	−0.41	0.684
Sex	Male	109 (67.28)	95 (65.52)	0.107	0.743
	Female	53 (32.72)	50 (34.48)		
BMI		22.32 (20.46–24.82)	22.49 (20.20–24.24)	−0.66	0.513
Location	Esophagogastric junction	79 (48.77)	32 (22.07)	23.624	< 0.001
	Non-Esophagogastric junction	83 (51.23)	113 (77.93)		
Tumor size, cm		5.30 ± 2.25	6.44 ± 3.19	3.11	0.002
Tumor differentiation	Well+Moderately differentiated	87 (53.70)	27 (18.62)	40.342	< 0.001
	Poorly differentiated+ Signet ring cell	75 (46.30)	118 (81.38)		
cT stage	T2 + T3	74 (45.68)	21 (14.48)	34.847	< 0.001
	T4	88 (54.32)	124 (85.52)		
cN stage	N0	57 (35.19)	10 (6.90)	35.889	< 0.001
	N+	105 (64.81)	135 (93.10)		
Borrmann classification	I + II	52 (32.10)	36 (24.83)	1.978	0.160
	III + IV	110 (67.90)	109 (75.17)		
Blood type	Type A	50 (30.86)	40 (27.59)	1.701	0.637
	Type B	50 (30.86)	39 (26.90)		
	Type AB	15 (9.26)	17 (11.72)		
	Type O	47 (29.01)	49 (33.79)		
CEA, ng/mL		6.5 ± 17.06	19.43 ± 99.23	1.30	0.198
CA724, U/mL		3.53 (1.50–10.63)	2.94 (1.88–12.83)	0.15	0.880
CA125, U/mL		13.98 ± 7.79	13.91 ± 9.20	−0.60	0.952
CA199, U/mL		37.45 ± 115.07	38.70 ± 97.23	0.09	0.930
Albumin, g/L		42.10 ± 3.52	41.24 ± 4.04	−1.72	0.086
PLT, 10^9^/L		212.03 ± 76.3	226.41 ± 90.12	1.31	0.192
Lymphocyte, 10^9^/L		1.58 ± 0.46	1.57 ± 0.47	0.07	0.945
PLR		134.00 (94.38–182.86)	136.99 (104.91–182.79)	0.88	0.382
Neutrophil cell, 10^9^/L		3.57 ± 1.33	3.64 ± 1.45	0.35	0.724
Monocyte, 10^9^/L		0.43 ± 0.16	0.40 ± 0.14	−1.37	0.173
NMR		8.23 (6.52–10.57)	8.97 (6.83–11.29)	0.92	0.360
NLR		2.22 (1.56–2.92)	2.28 (1.68–2.78)	0.18	0.860
MSI	H	13 (8.02)	9 (6.21)	0.380	0.538
	S/L	149 (91.98)	136 (93.79)		
Smoking history	yes	44 (27.16)	69 (47.59)	13.724	< 0.001
	no	118 (72.84)	76(52.41)		

*BMI* Body Mass Index; *CA125* Carbohydrate antigen 125; *CEA* Carcinoembryonic antigen; *CA724* Carbohydrate antigen 724; *CA125* Carbohydrate antigen 125; *CA199* Carbohydrate antigen 199; *PLT* Platelets; *PLR* platelet to lymphocyte ratio; *NMR* neutrophil to monocyte ratio; *NLR* neutrolphil to lymphocyte ratio; *MSI* microsatellite instability

**Fig. 2:**
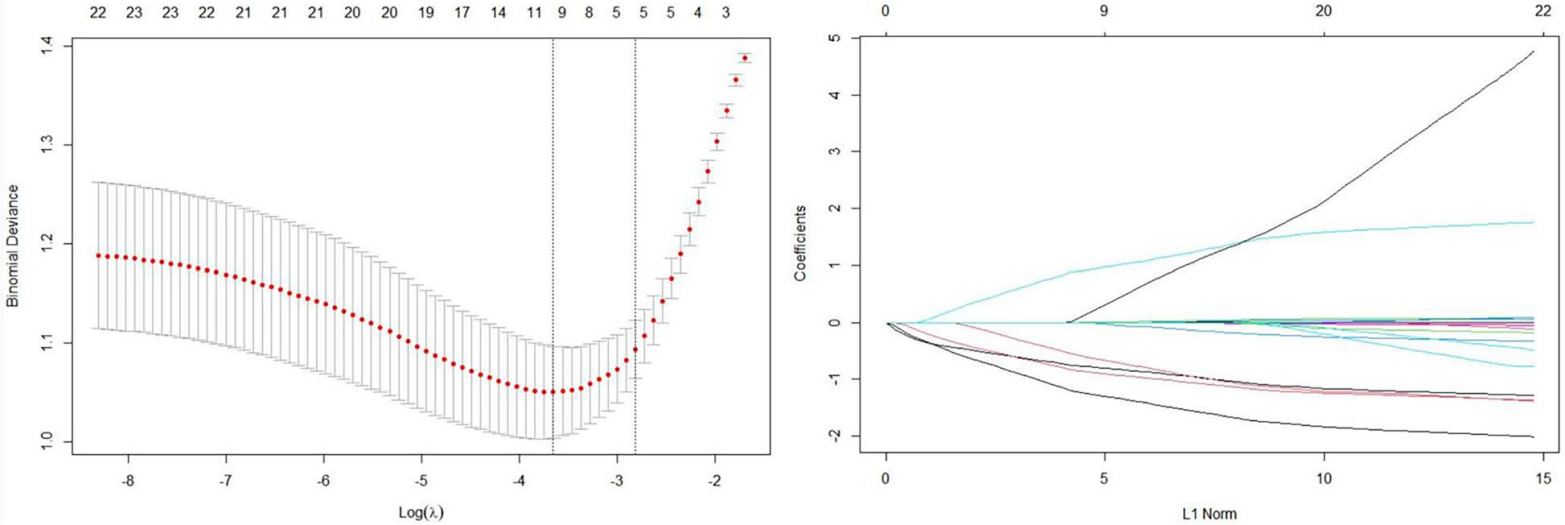
Texture feature selection using the least absolute shrinkage and selection operator (LASSO) binary logistic regression model

**Fig. 3:**
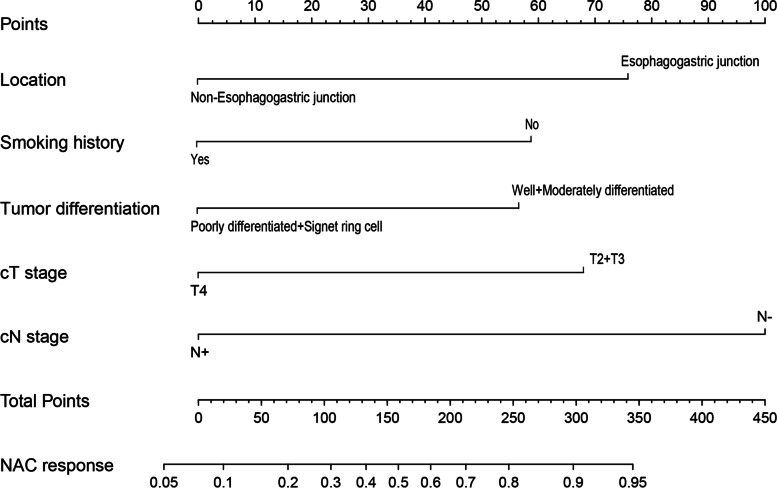
Nomogram for predicting response to neoadjuvant chemotherapy

**Fig. 4:**
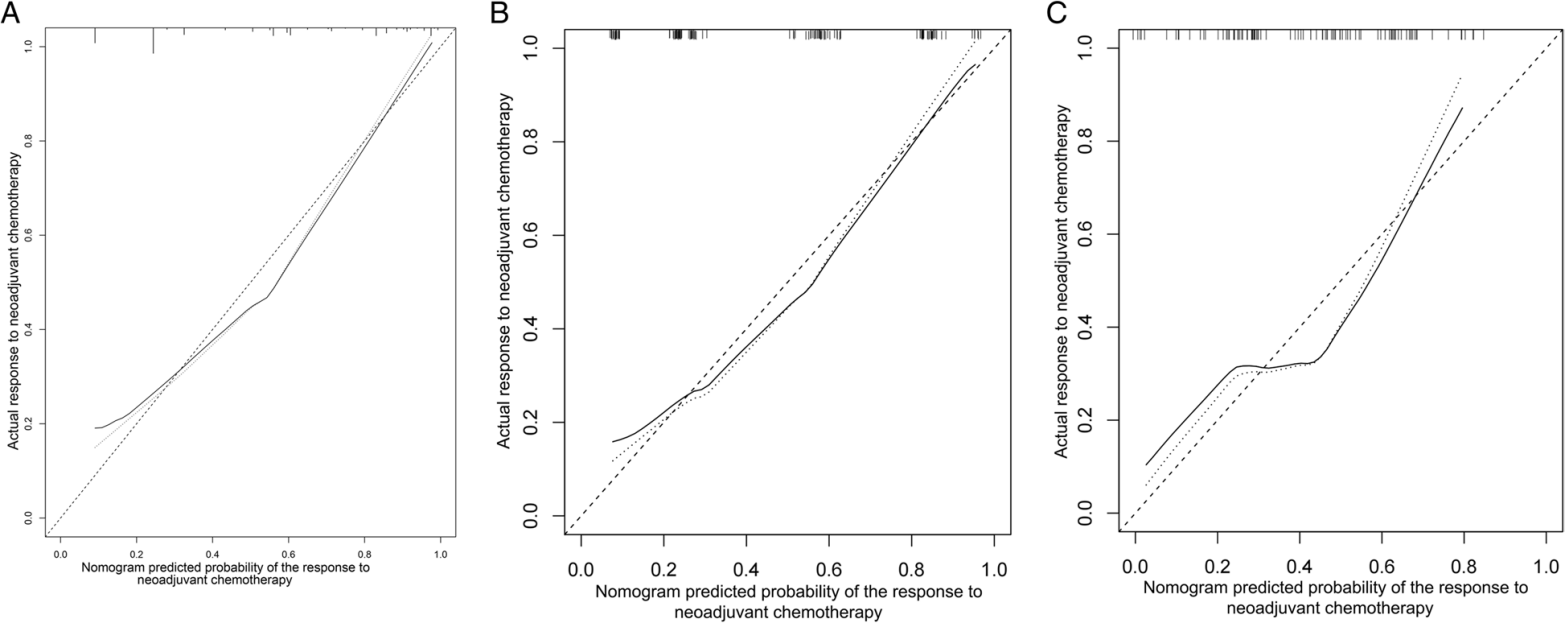
Calibration curve for the nomogram model in the training (A), internal validation (B) and external validation sets (C)

**Fig. 5:**
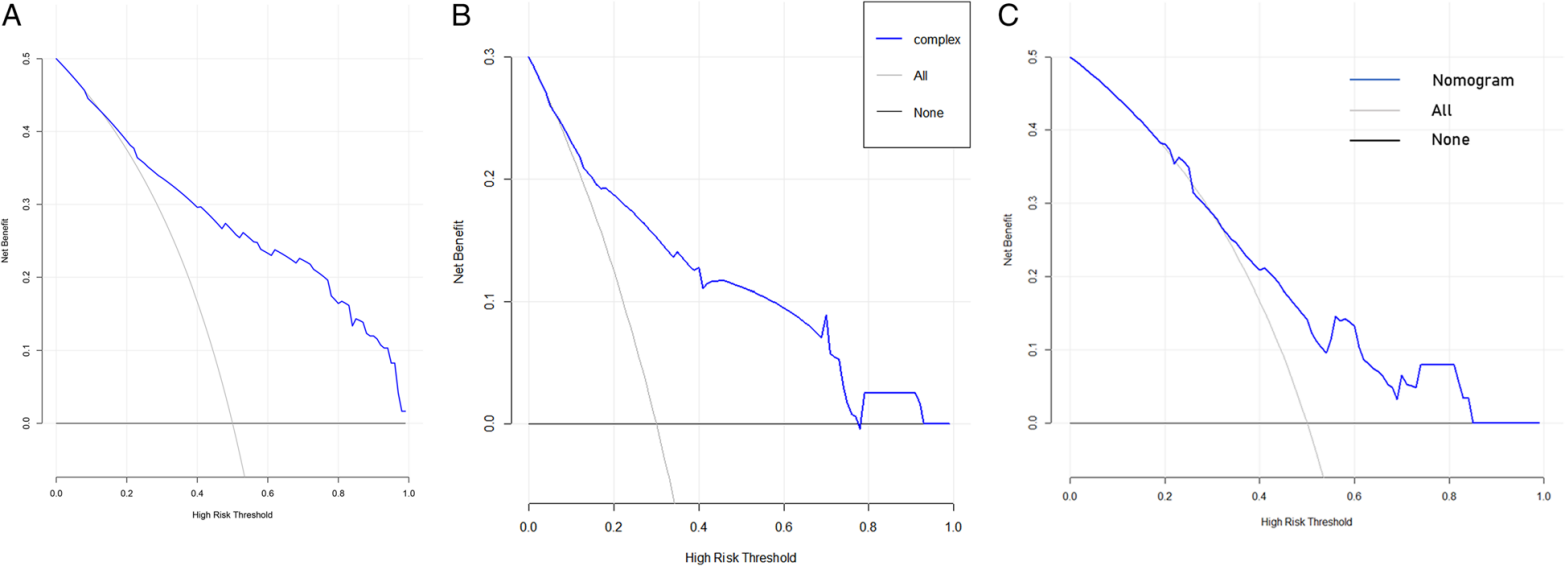
DCA analyzed clinical utility of the nomogram in the training (A), internal validation (B) and external validation sets (C). The y-axis represents net benefits and the x-axis measures threshold probability (Pt). The horizontal solid line indicates the advantage for patients not receiving NAT, the oblique solid line represents the advantage for patients receiving NAT and the diagonal dotted line (nomogram) indicates survival on the basis of nomogram scores to resolve whether a patient should receive NAT. A treatment strategy was superior if it had the highest value compared to other models, including two simple strategies, such as performing NAT for all patients (sloping solid line) or performing primary surgery first (horizontal solid line)

To address the problem of multicollinearity upon regression, we examined distribution coefficient in the logistic regression using LASSO with the elastic net penalty. Lasso analysis excluding tumor size variable showed that five prediction factors were incorporated into the eventual model, including differentiation degree, tumor location, clinical T stage, Clinical N stage and Smoking history (Fig. 2 and Table 3). Afterwards, the nomogram incorporating those aforementioned factors was constructed (Fig. 3), and its CI values were determined to be 0.842, 0.806 and 0.760 for training, internal and external validation sets, respectively. It indicated the good predicting ability of our as-constructed nomogram. For our NACT nomogram, its calibration curve was in good consistence between predicted and actual results for primary cohort in training, internal and external validation sets (Fig. 4). The value of the nomogram and its use in the clinic was evaluated by DCA to assess our nomogram for its significance and clinical use, which are displayed in Fig. 5.

**Table 3:** Result of multivariable analysis in the training set. As revealed by lasso analysis excluding tumor size variable, five prediction factors were incorporated into the eventual model, which were differentiation degree, tumor location, Clinical T stage, Clinical N stage and Smoking history

Items	Regression coefficient	Exp(B)	95%CI	*P* value
location	−1.561	0.212	0.103–0.438	< 0.001
Tumor differentiation	1.103	3.113	1.510–6.418	0.002
cT stage	1.361	3.852	1.828–8.119	< 0.001
cN stage	2.040	7.621	2.838–20.463	< 0.001
Smoking history	1.129	3.226	1.606–6.477	0.001
C-index	0.842

## Discussion

Surgical resection remains the mainstream therapeutic strategy for GC. Over 60% of cases enter the advanced stage when they are diagnosed, causing the poor curative treatment rate of GC, and it is necessary to establish the effective approach to increase the curative treatment rate [21].

It has been previously suggested that surgical treatment can induce the transformation of cancer cells to drug-resistant clones and increase cancer cell growth by elevating cancer growth-stimulating factor secretion. Cancer cells are low at the early stage and the proliferation rate is high, while the doubling time is relatively short, and cancer cells show high sensitivity to chemotherapeutics [22]. As a result, chemotherapeutics administered prior to surgical treatment can suppress primary cancer and inhibit growth-stimulating factor production in tumor cells, which is efficient in micrometastasis. Early application of chemotherapy resulted in a low number of drug-resistant cells [23], emphasizing the importance of NACT.

At present, preoperative NACT has attracted great attention, and it functions to assist surgeons in reducing tumor stage and size, removing micrometastasis, mitigating associated symptoms, increasing radical treatment rate and decreasing relapse rate after surgery. However, a small portion of cases cannot respond to chemotherapeutics and cannot obtain benefits from NACT, giving rise to cancer development and delayed surgical treatment. Approximately 15% of cases who receive NACT are associated with a higher risk of disease progression [24]. In addition, cases usually experience adverse reactions following NACT, like toxicity to the liver, kidney, and heart, leading to higher mortality and complication rates at the time of surgical treatment. As a result, it is important to predict NACT response. This study focused on identifying pre-treatment parameters for predicting NACT response, aiming to lay a certain foundation to perform personalized treatment for GC. For cases developing favorable NACT response, NACT can be applied. Otherwise, comprehensive therapy or surgery can be applied early.

According to this study, advanced GC patients receiving NACT achieved an effective rate (including CR and PR) of 52.6%, indicating that just some cases obtained benefits from NACT and suggesting that it was important to predict NACT response. Univariate analysis showed that we identified tumor site, infiltration depth, lymph node metastasis, tumor differentiation, smoking status and tumor size as the factors to predict chemotherapy response, while the tumor size factor was excluded by LASSO logistic regression analysis due to a linear relationship between tumor size and tumor invasion depth. Using those remaining five factors, the nomogram was established to predict chemotherapy response prior to gastrectomy plus LND.

The results of the studies on response to neoadjuvant chemotherapy have been inconsistent. Data from studies performed in Japan show that locally advanced GC cases receiving lymphadenopathy, borrmann IV and tumor diameter > 7 cm obtain the most benefits from NAT [25–27]. However, our research showed the opposite result, indicating that patients with lower infiltration depth, lower differentiation and without lymph node metastasis had a better response to neoadjuvant chemotherapy, conforming to the result of Wang et al. [7]. In a retrospective cohort study performed in Germany, 410 cases were enrolled, and it was found that tumor located in the top 2/3 stomach responded well to NAT [28]. Similar results were reported in Li et al.’s study [29], conforming to our findings that cases whose tumor was located at the esophagogastric junction obtained an increased chemotherapy response rate (71.08%) compared with those whose tumor was not located at the esophagogastric junction (42.18%), with no significant difference (*P* < 0.05).

It is found that smoking attenuates the oxidative burst of neutrophils and monocytes and enhances chemotaxis [30], and patients with a smoking history are adversely associated with response to NAC in bladder cancer and ovarian cancer [31–33]. In addition, the study also indicated that the number of neutrophils in patients with smoking history was higher than that in patients without smoking history (P < 0.05, data not shown) and smoking was an independent factor associated with a poorer chemotherapy response. In tumor microenvironment (TME), platelets, lymphocytes and neutrophils exert critical roles in cancer metastasis and development because chemokines and inflammatory cytokines are produced [34–39]. The increased neutrophil/platelet counts and the decreased lymphocyte count, generally suggests the damaged immune activity and higher inflammatory response, thereby promoting cancer cell growth, LNM, invasion and distant metastasis. However, this study suggested that inflammatory factors including platelets, lymphocytes or neutrophils were not the factors independently predicting chemosensitivity.

Inconsistent with previous studies [26], this study suggested the superior NACT reactivity among cases with low T stage (T2, T3) and N stage (N0) compared with advanced T stage (T4) and N stage (N+). Meanwhile, our study also discovered that 39.08% of patients had a descending lymph node. Some scholars consider that positive lymph node indicates a poor prognosis, and the elimination of nodal micrometastasis is the reason for NACT [40]. Because neoadjuvant chemotherapeutic drugs have severe side effects, which bring adverse reactions among cases and damage nervous, hematological and digestive systems [8]. As a result, selecting the best therapeutic strategy for diverse cases is of great significance. Therefore, we should focus on neoadjuvant chemotherapy for patients with early TNM stage, while in NACT-insensitive cases, additional NACT regimens should be applied, including FLOT (fluorouracil plus leucovorin, oxaliplatin, and docetaxel), and it achieves higher OS than ECX [41]. In addition, another method for these patients is early surgical treatment after chemotherapy.

Numerous recent studies mainly emphasize the association of MSI with prognosis of GC cases. In this work, MSI was not correlated with the response to NAC.

A nomogram that predicted NACT response was constructed, and its CI value was as high as 0.767 [8]. In this work, the CI value was 0.842 by our nomogram, indicating that our nomogram made better performance than previous reports in predicting survival.

Certain limitations should be noted in the present work. At first, our findings were possibly biased because of the retrospective nature. The C-index of this study was too high, which might be due to that the model was overfitted to the study’s cohort. Second, since many cases were recruited into this work within 2 years, we were unable to obtain sufficient survival data to explore how chemosensitivity and predictors affected OS. At first, some previous research showed that tumor markers such as CEA, CA199, CA724 and CA125 were implicated in chemosensitivity, whereas others did not [7, 8, 42–45]. Our study suggested that tumor markers were not the independent predictors for chemosensitivity. Thus, more large and high-quality studies should be performed to address these issues. In addition, we eliminated 117 cases out of this study due to the insufficient data. Multiple imputation may serve as a favorable approach for supplementing the insufficiency of data. However, among those 117 cases, imaging data from numerous cases before chemotherapy were obtained in other hospitals, making it impossible to evaluate accurate chemotherapeutic response. Therefore, these studies were eliminated during data extraction.

## Conclusions

Five risk factors were significantly associated with NACT effectiveness, including clinical T stage, clinical N stage, differentiation, tumor location, and smoking history. The as-constructed nomogram exhibited the satisfying effect on predicting NACT response, and it might be adopted for selecting the best strategy to treat advanced GC cases by gastrointestinal surgeons. For example, neoadjuvant chemotherapy is not recommended for patients with gastric cancer who have lesions at the esophagogastric junction, positive LNM, poorly differentiation, smoking history, and T4 infiltration depth.

## Data Availability

All data and materials supporting our findings can be obtained from corresponding author upon request. Data were public for the sake of privacy and ethical restrictions.

## Abbreviations

NAC: neoadjuvant chemotherapy
GC: Gastric cancer
BMI: Body Mass Index
OS: overall survival
DFS: disease-free survival
CEA: Carcinoembryonic antigen
CA125/199/724: Carbohydrate antigen 125/199/724
PLT: Platelets
PLR: platelet to lymphocyte ratio
NLR: neutrolphil to lymphocyte ratio
NMR: neutrophil to monocyte ratio
MSI: microsatellite instability.
